# The physiological and pathological biophysics of phase separation and gelation of RNA binding proteins in amyotrophic lateral sclerosis and fronto-temporal lobar degeneration

**DOI:** 10.1016/j.brainres.2018.04.036

**Published:** 2018-08-15

**Authors:** Peter St George-Hyslop, Julie Qiaojin Lin, Akinori Miyashita, Emma C. Phillips, Seema Qamar, Suzanne J. Randle, GuoZhen Wang

**Affiliations:** a Cambridge Institute for Medical Research, Department of Clinical Neurosciences, University of Cambridge, Cambridge CB2 0XY, UK; b Tanz Centre for Research in Neurodegenerative Diseases, and Departments of Medicine, Medical Biophysics and Laboratory Medicine and Pathobiology, University of Toronto, Toronto, Ontario M5S 3H2, Canada; c Department of Physiology, Development, and Neuroscience, University of Cambridge, Cambridge CB2 3DY, UK

**Keywords:** ADMA FUS, asymmetrically di-methylated arginine FUS, CHOP, C/EBP homologous protein gene, DDX4, DEAD box helicase 4, EWS, Ewing sarcoma protein, fALS, familial amyotrophic lateral sclerosis, FTLD, frontotemporal lobar degeneration, FUS, fused in sarcoma protein, hnRNP, heterogeneous nuclear ribonucleoprotein, PTM, post-translational modification, PY-NLS, proline tyrosine nuclear localisation signal, QGSY, glutamine glycine serine and tyrosine repeats motif, RGG, arginine glycine glycine repeat motif, RRM, RNA recognition Motif, SMN, survival motor neuron, TAF15, TATA box binding protein 15, TDP-43, transactive response DNA binding protein 43, TNPO1, transportin 1/karyopherin β2, Amyotrophic lateral sclerosis, Arginine methylation, Cation-pi interactions, Frontotemporal dementia, Phase separation, RNA binding proteins

## Abstract

Many RNA binding proteins, including FUS, contain moderately repetitive, low complexity, intrinsically disordered domains. These sequence motifs have recently been found to underpin reversible liquid: liquid phase separation and gelation of these proteins, permitting them to reversibly transition from a monodispersed state to liquid droplet- or hydrogel-like states. This function allows the proteins to serve as scaffolds for the formation of reversible membraneless intracellular organelles such as nucleoli, stress granules and neuronal transport granules. Using FUS as an example, this review examines the biophysics of this physiological process, and reports on how mutations and changes in post-translational state alter phase behaviour, and lead to neurodegenerative diseases such as amyotrophic lateral sclerosis and frontotemporal lobar degeneration.

## Highlights

• Some intrinsically disordered proteins undergo reversible phase separation/gelation.
• Reversible phase separation/gelation underpins function of membraneless organelles.
• fALS-FUS mutations increase propensity of FUS to form highly stable condensates.
• Changes in arginine methylation and FUS chaperones in FTLD-FUS have similar effects.
• Stable fibrillar condensates sequester cargo and impair RNP granule function.

## Graphical abstract

**Figure:**
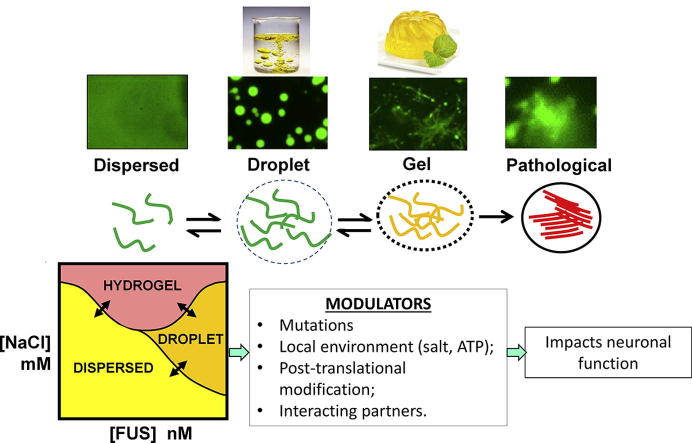

The discovery of missense mutations in multiple RNA binding proteins, such as fused in sarcoma (FUS) (Kwiatkowski et al., 2009, Vance et al., 2009), transactive response DNA binding protein 43 (TDP-43) (Kabashi et al., 2008, Sreedharan et al., 2008), heterogeneous nuclear ribonucleoprotein (hnRNP) A1 and A2/B1 (Kim et al., 2013), in patients with familial forms of amyotrophic lateral sclerosis (ALS) and frontotemporal lobar degeneration (FTLD), has opened up a new and highly productive avenue of research into the pathobiology of ALS and FTD. It has also shed light on a previously poorly recognised field of cell biology, namely the role of intrinsically disordered proteins in the formation and function of membraneless intracellular organelles such as nucleoli, stress granules, ribonucleoprotein (RNP) granules, P-bodies and neuronal transport granules.

A common neuropathological feature of ALS and FTLD associated with mutations in RNA binding proteins is the deposition of visible aggregates of the corresponding proteins in the nucleus and/or cytoplasm of neurons and glia (Mackenzie and Neumann, 2016, Neumann et al., 2006, Neumann et al., 2009, Rademakers et al., 2012). These protein aggregates display subtle differences in their staining and biochemical characteristics compared to conventional amyloid β-sheet rich aggregates associated with other neurodegenerative diseases such as Aβ, tau and α-synuclein. For instance, they stain poorly with amyloidophyllic dyes such as thioflavin T, they contain little β-sheet content on circular dichroism, and they are partially soluble in urea (Mann and Snowden, 2017, Neumann et al., 2006, Neumann et al., 2009, Rademakers et al., 2012). Because these assemblies are distinguishable from classical amyloids, it has become of intense interest to determine the underlying mechanisms of the neurotoxicity of these aggregates (Bowden and Dormann, 2016, Coady and Manley, 2015, Kim and Taylor, 2017, Lagier-Tourenne et al., 2010).

This review focuses on recent work suggesting that many of these soluble RNA binding proteins possess the unique biophysical property of being able to *reversibly* transition between a dispersed (mixed) state; a phase-separated state as liquid protein droplets suspended within a liquid; or in a gelled state as soft hydrogels somewhat similar to jelly dessert. This review will also describe work that has shown that disease associated mutations and disease associated changes in the post-translational modifications (PTM) in some of these proteins dramatically alter this process, resulting in formation of stable condensed gelled states that are not easily reversible by the cell. The physics of separation of polymers into droplets and gels are well known in materials science, but were not widely thought about in biological science until very recently. As a result, the terminology used in this field is in a state of flux, although efforts are being made to assemble a uniform nomenclature for these processes. Currently, the biophysical condensation process of polymer phase separation from a dispersed state into liquid droplet suspended in a liquid has been termed “liquid: liquid phase separation” or “coacervation”. The phase separation from a dispersed or liquid droplet state into a gelled state has been termed “gelation”. A more general term that is often applied to these alternate biophysical states is “condensate” (Shin and Brangwynne, 2017). However, the reader is alerted to the fact that many other terms, such as “de-mixing” are also frequently used.

## 1 Liquid droplets and hydrogels as the building block of membraneless organelles

The unique biophysical property of reversible condensation of RNA binding proteins associated with ALS and FTD (and also of a growing list of other proteins) turns out to be crucial for the formation of a subset of intracellular organelles which lack limiting membranes. These membranous organelles include nucleoli, stress granules, neuronal transport granules, and postsynaptic densities to name a few. Most intracellular organelles possess a limiting membrane which, because of their very different biophysical properties and ability to bind histological dyes, facilitated their visualisation by early investigators using conventional light microscopy. In contrast, intracellular organelles that lack limiting membranes, and that have biophysical properties very similar to those of the surrounding cytoplasm, were difficult to visualise by conventional light and electron microscopy. As a result, their authenticity was initially questioned. However, recent advances in live cell imaging methods including the use of fluorescent tags and tools such as optical tweezers, have made it clear that membraneless organelles such as nucleoli, stress granules and RNP granules are indeed authentic physiological structures (Banani et al., 2017, Brangwynne et al., 2009, Hyman et al., 2014).

**Fig. 1:**
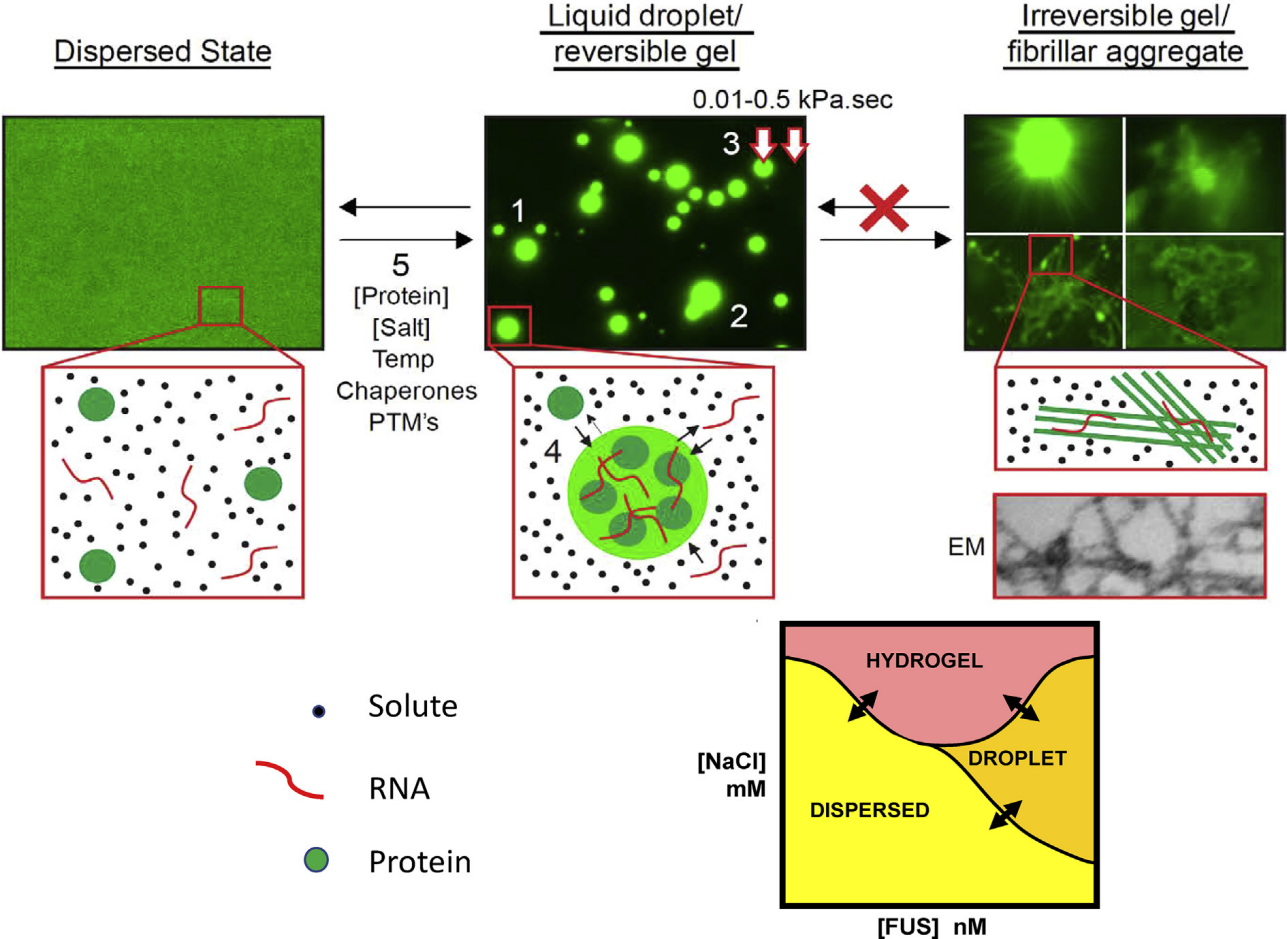
Cartoon of phase separation. In the dispersed state, protein scaffolds (green circles) and cargo/client RNA molecules (red lines) are intermixed with solute molecules (black circles). Under appropriate conditions, protein scaffolds can phase separate to form a liquid droplet enriched in the scaffold protein and client RNA. These condensates display several features of liquid droplet including: (1) spherical shape; (2) the ability to fuse with each other; (3) viscosity that is similar to or only slightly increased relative to the viscosity of water (left red arrow denotes the FUS droplet; right red arrow denotes the solute; the figure above denotes the approximate range of viscosity difference in kilo Pascal seconds; (4) ability for both client and scaffold molecules to partition in and out; and (5) the ability to relax/disassemble back to dispersed state. They can also further condense into fibrillar aggregates. The relationships between hydrogel, droplet and dispersed states can be depicted in a classical phase diagram with conversion between each of the states.

Generally, but with some exceptions, membraneless organelles have the biophysical characteristics expected of liquid droplets suspended in an immiscible liquid. They are often spherical. They fuse into larger droplets when they contact each other (Fig. 1). They display viscosity similar to that of water. The component polymers that make the 3D structure of the condensate (“scaffolds”) are generally not bound to each other by strong covalent forces. As a result, the component polymers of these membraneless organelles often display rapid exchange with the surrounding solute. Other molecules in the solute which are capable of binding to the scaffold proteins can also rapidly partition between the solute and the droplet/gel phase by diffusion. The efficiency of the partitioning of these “client” or “cargo” molecules reflects a variety of factors including the affinity of the scaffold protein for the client (e.g. affinity for binding of specific RNAs to the RNA Recognition Motifs of RNA binding proteins). Finally, it is important to note that unlike conventional protein complexes, these membraneless organelles are not in stable, steady-state equilibrium states. Instead, they are “metastable” assemblies whose biophysical state is fragile, and is easily perturbed by external modulators. Such external modulators include post-translational modifications and remodelling by ATPase-driven chaperones and disaggregases (see below).

Conceptually, the simplest membraneless intracellular organelle would be a “homotypic” droplet composed of a single phase separating polymer. However, in practical terms, most cellular membraneless organelles are more complex. As an example of morphological complexity, stress granules have been described as having a semisolid gel-like cores (Jain et al., 2016) or multiple, distributed small nanocores (Niewidok et al., 2018), a difference which might reflect differences between fixed and unfixed samples and/or in cell type of origin. Other membraneless organelles such as nucleoli, are also composed of multiple distinct liquid droplets within a larger encompassing droplet (Feric et al., 2016), which can be re-created experimentally (Schmidt and Rohatgi, 2016). Membraneless organelles are also biochemically complex, often containing hundreds or even thousands of RNA and protein components (Fritzsche et al., 2013, Jain et al., 2016, Markmiller et al., 2018, Youn et al., 2018). Moreover, the component proteins are generally not specific to a single type of membraneless organelle, with many proteins being components of several nosologically distinct membraneless organelles (Fritzsche et al., 2013, Jain et al., 2016, Markmiller et al., 2018, Youn et al., 2018). The composition of some complexes can differ under different contexts (normal versus disease) or cell type (Markmiller et al., 2018, Youn et al., 2018). Nevertheless, despite this morphological and biochemical complexity each of these condensates are still governed by, and predicted by, the same general biophysical rules that govern the assembly of simpler complexes, which are discussed below.

## 2 Functional implications of physiological phase separation and gelation

The biophysical properties of membraneless organelles, which allow reversible transition between dispersed, liquid droplet and gel-like states, confer the ability of these organelles to perform a wide range of biological functions such as transport, storage and physical colocalization of components of intracellular signalling or metabolic machinery. For instance, stress granules allow sequestration of cargo (i.e. key translationally-stalled mRNA transcripts in the case of stress granules) during cellular stress (Protter and Parker, 2016). Neuronal transport granules sequester and transport key cargo elements involved in regulated local protein translation in axon terminals and dendrites (Holt and Bullock, 2009, Holt and Schuman, 2013, Shigeoka et al., 2016). Other membraneless organelles, such as the post-synaptic density in neuronal dendritic spines allow the assembly of specific reaction components necessary for signalling downstream of the post-synaptic membrane (Zeng et al., 2016). The diversity of the cell types harbouring membraneless organelles, and the diversity of their functions, emphasizes the critical role played in cell biology by membraneless organelles and by the components which underpin their ability to phase separate.

## 3 Composition and structure of proteins that form liquid droplets

Most soluble intracellular proteins (e.g. classical enzymes) exist as uniformly mixed, dispersed solutions of either monomeric protein or higher order complexes. These proteins generally fold into a limited number of well-defined, well-ordered three-dimensional shapes (“folds”) that confer the functional properties of these proteins. However, recent work has identified an increasingly large group of soluble proteins that can exist in a soluble dispersed state, but that can also transition into a separate physical state in which the protein exists in liquid droplets of concentrated protein suspended within the liquid solvent (i.e. the cytoplasm/nucleoplasm). Some of these proteins can also condense into jelly-like states, which are referred to as “hydrogels”. This ability to phase separate is also held by other biological polymers (e.g. some RNAs) (Jain and Vale, 2017) and by man-made polymers.

**Fig. 2:**
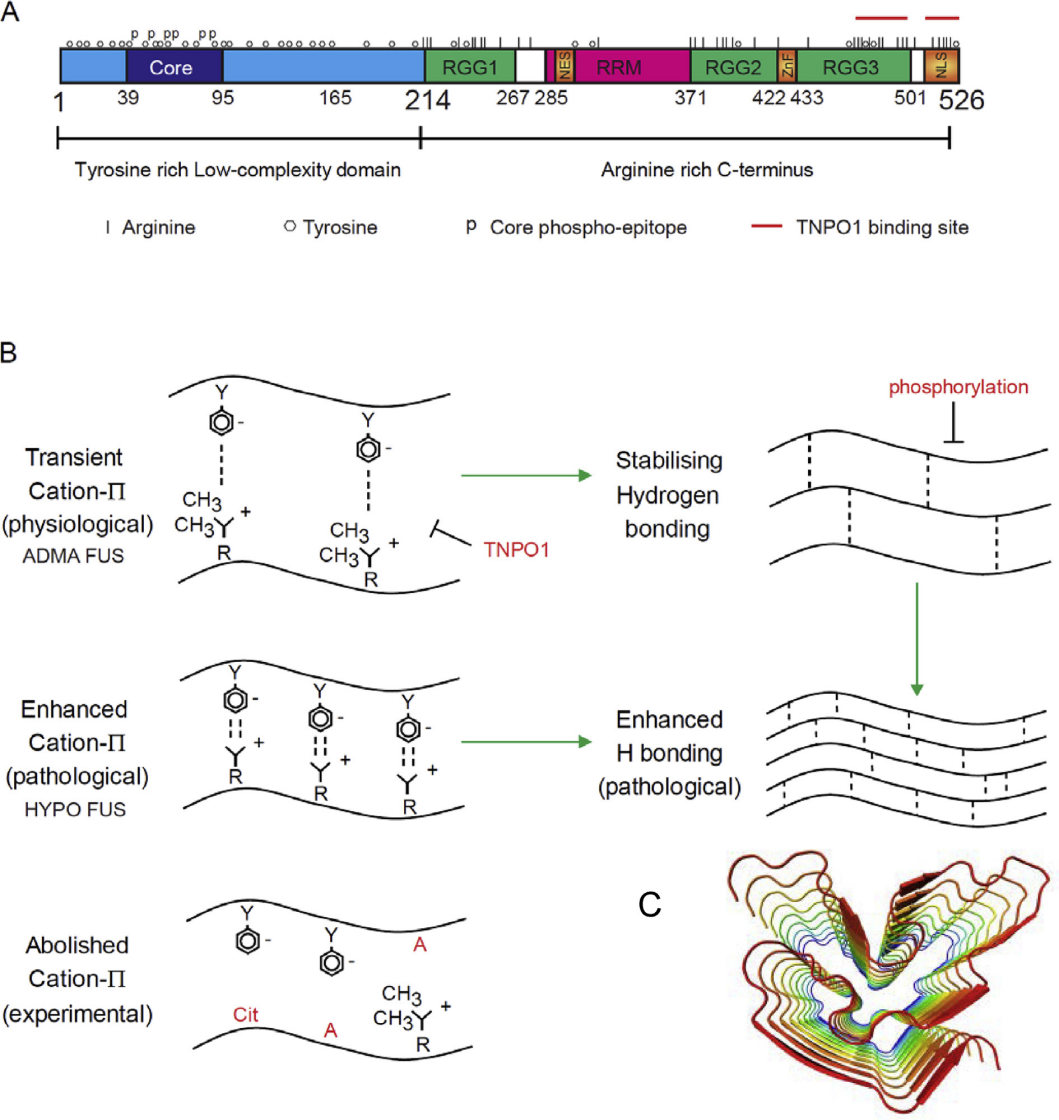
A. Cartoon of FUS protein topology, showing the tyrosine rich low complexity (LC) domain and the arginine rich C-terminus comprised of structured arginine glycine glycine motifs (RGG) and RNA recognition motifs (RRM), as well as the C-terminal, atypical proline tyrosine nuclear localisation sequence (PY-NLS). B. Cartoon diagrams of arginine: tyrosine cation- π interactions. These interactions can be enhanced by demethylation of arginines. They can be abolished by conversion of arginine to citrulline. Further stabilising intermolecular hydrogen bonding between β-sheet motifs stabilise liquid droplet and hydrogel condensates, and if excessive, the formation of irreversible fibrillar gels. C. Packing of e β-sheet motifs within the LC domain can form fibrillar structures with many similarities to classical amyloid fibrils (adapted from Murray et al., cell 171:165, 2018.

**Fig. 3:**
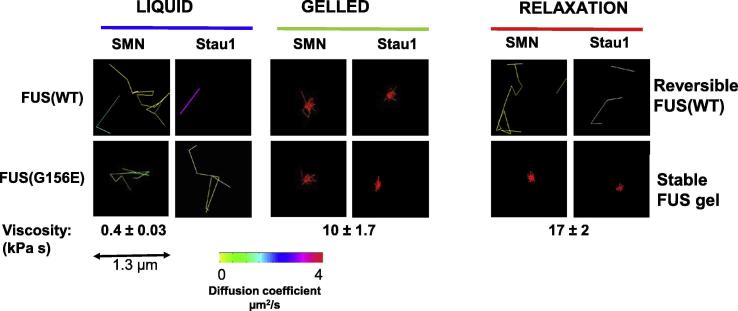
Phase separation and gelation can result in sequestration of client and cargo elements, such as other ribonucleoproteins and RNAs. This can be reversed upon relaxation/melting of the droplet or gel. *Top row*: shows single particle tracking of individual survival motor neuron protein and staufen-1 protein molecules in wild-type FUS dispersed assemblies (*left column*) and in liquid droplet/hydrogel condensates (*middle column*). The sequestration of the client/cargo element can be reversed by relaxation of the condensate a liquid droplet or dispersed state (*right column*). *Bottom row* depicts irreversible sequestration of client molecules in fALS-FUS mutant FUS condensates. In all images the length of the single molecule path is depicted and the diffusion coefficient for movement in that path is colour-coded according to the heat map. The viscosity of the condensates is depicted below the images (kPa·s). Adapted from Murakami et al., Neuron 88:678, 2015.

A common feature of these phase-separating proteins is the presence of at least one domain composed of repetitive stretches of amino acids with polar side chains (glycine, glutamine, asparagine and serine), nonpolar side chains (proline), positive side chains (arginine, lysine), negative side chains (aspartate, glutamate) or aromatic side chains (phenylalanine, tryptophan and tyrosine). Hydrophobic residues on the other hand, are typically underrepresented. These amino acids are often arranged in imperfect, short repetitive motifs (e.g. enrichment of glutamine glycine serine tyrosine (“QGSY”) repeats in the N-terminus of FUS) (Fig. 2A). Because of their reduced amino acid diversity, these domains are often referred to as “low complexity” (LC) domains. Proteins or protein domains with these features typically do not fold into a single, well-defined three-dimensional structure, and are thus frequently also described as “intrinsically disordered” proteins or domains (See Fig. 3).

## 4 Biophysics of liquid-liquid phase separating polymers

In the last several years the biophysics of phase-separated intracellular structures has been probed using both theoretical and experimental approaches (Banani et al., 2017, Bergeron-Sandoval et al., 2016, Brangwynne et al., 2015, Pak et al., 2016, Shin and Brangwynne, 2017, Wei et al., 2017). The phase transition of soluble polymers between dispersed, liquid droplet and hydrogel-like phases, represents a well-understood thermodynamic event in which an initial uniform mixture of dispersed polymers (e.g. single proteins, single RNA, or mixtures of RNA and protein) finds a lower free energy state when the components separate into one or more distinct phases (e.g. a polymer-rich liquid droplet phase and a separate polymer-depleted solute phase) (Fig. 1). This situation typically occurs when each of the component molecules in the mixture “prefers” interactions with itself over interactions with the solute molecules. In the simplest instance, this might result in the partitioning of the protein or RNA polymer into a polymer-rich droplet (where its concentration is then much higher than in the initial dispersed liquid state) suspended in a polymer-depleted liquid solvent (Fig. 1). However, other more complicated phase separations can be imagined. For instance, two protein molecules, or protein plus RNA molecules, might cooperatively interact with each other to form a liquid droplet enriched in both components, suspended in liquid solute that is depleted in both. This latter situation, as discussed below, sets up a circumstance where the relative stoichiometries of the components can modulate the propensity to phase separate.

The forces (or “preferred interactions”) that drive liquid–liquid phase separation include both long-range forces that might initiate the phase separation, and intermediate-range forces that might subsequently stabilise the assembly once polymer molecules have begun to form a network with each other. The longer-range forces are typically charged interactions (e.g. glutamate – arginine), whereas the intermediate-range forces include directional interactions between positively charged residues and aromatic residues (for instance cation-π interactions between the positively charged side chains of arginines or lysines with the free electrons in the aromatic rings of tyrosines, tryptophans or phenylalanines). Other intermediate-range interactions include dipole interactions between glycine, glutamine, asparagine and serine residues, as well as π-π interactions formed by stacking of aromatic rings or between the guanidino moiety of arginines and the rings of aromatic amino acids (Vernon et al., 2018). Most low complexity domains are relatively depleted in hydrophobic amino acids, but where they do occur, they can manifest by phase separation that occurs upon increasing temperature, rather than phase separation with decreased temperature, which is typically observed for most polymer systems (Jiang et al., 2015, Reichheld et al., 2017). Finally, weak short range intermolecular hydrogen bonding between β-sheet motifs occurs in liquid droplet, hydrogel and in denser fibrillar condensates (Hughes et al., 2018, Murray et al., 2017, Qamar et al., 2018). The denser fibrillar condensates possess more extensive β-sheet driven intermolecular hydrogen bonding, and often result in stable pathological fibrillar assemblies that cannot easily be reversed by the cell (and are often therefore referred to as “irreversible” condensates).

Crucially, the interactions driving phase separation are unlike the lock-and-key interactions of well-folded proteins, which rely on precise three-dimensionally specific point-to-point interactions. In phase separating proteins, the interactions build upon the disordered state and the repetitive sequence motifs that are characteristic of the LC domains of these proteins. Residues within these repetitive sequences can be considered as interaction points (often referred to as “nodes”, although the use of such network terminology is not universally preferred). These residues/nodes permit the formation of networks of intra- and inter-molecular interactions within and between phase-separating polymers (Brangwynne et al., 2015, Harmon et al., 2017, Wei et al., 2017). The strength of this overall network is therefore driven by: i) the number (or “valence”) of interacting nodes (e.g. the number of arginine: tyrosine cation-π or π-π interactions); and ii) the strength of the interaction at each node. As an example of this multivalency, the LC domain of FUS contains 27 tyrosines, which can form cation-π interactions with 37 arginines mostly found in the structured C-terminus (Qamar et al., 2018) (Fig. 2A). Phase separation is not dependent on any individual FUS tyrosine or arginine residue (Lin et al., 2017, Qamar et al., 2018). Conversely, phase separation is enhanced by selective mutagenesis which introduces additional arginine residues, and this enhancement is dependent on the number of extra arginine residues, rather than their position. Indeed, the exact positions of the deleted or added tyrosine residues (in the LC domain) or arginine residues (in the C-terminal domain) seems irrelevant, emphasising that precise three-dimensional spatial orientation of the interactions is less critical than the valence of the interactions (Qamar et al., 2018).

Because liquid: liquid phase separation is primarily driven by *multiple* inter- and intra-polymer interactions that are not highly restricted to specific three-dimensional orientations, this allows phase separation to be tuned simply by manipulating number of available interactions/nodes (e.g. by posttranslational modifications). Thus, as described below, the number of available interactions/nodes in FUS can be changed by altering the post-translational state of the key residues (e.g. arginine methylation or serine phosphorylation) or by shielding the key residues/nodes by cloaking them with interacting chaperones (e.g. transportin 1 – TNPO1) (Deng et al., 2014, Guo et al., 2018, Hofweber et al., 2018, Monahan et al., 2017, Murray et al., 2017, Qamar et al., 2018, Yoshizawa et al., 2018).

Condensation into hydrogels and into stable fibrillar condensates (i.e. “gelation”) likely follows somewhat similar rules, but in a time- and concentration-dependent manner in which intermolecular hydrogen bonding of β-sheet domains becomes increasingly important, especially in fibrillar condensates. However, the specific details of this process are currently the topic of intense scrutiny. Work on fragments of FUS using electron microscopy, solid state nuclear magnetic resonance and x-ray diffraction studies of fibrillar condensates reveals several important differences from classical amyloids including: i) short β-sheet domains (often <5 residues); ii) a paucity of hydrophobic residues; iii) high prevalence of hydrophilic residues; iv) the presence of “kinks” at glycine, proline or aromatic residues which preclude long β-sheets and thus minimize the ability to make stable, steric zippers characteristic of conventional amyloids (Hughes et al., 2018, Murray et al., 2017). These motifs (e.g. residues ^37^-SYSGYS-^42^ and ^54^-SYSSYGQS-^61^ in the putative amyloid-core domain of FUS FUS) have recently been termed “low-complexity aromatic-rich kinked segments” or LARKS (Hughes et al., 2018) (Fig. 2B and C). However, while these emerging results are exciting, they should be interpreted with caution. First, the fragments studied in these experiments are not physiological. Second, there are amyloid forming proteins (e.g. yeast prion proteins such as Sup35) that have similar amino acid content (King et al., 2012).

## 5 Mathematical modelling and complex membraneless organelles

The fundamental biophysical processes underlying these phase separations can be reasonably well modelled mathematically based upon Flory Huggins models (which consider a network of the molecules interacting on a lattice) (Das et al., 2015, Flory, 1942, Huggins, 1942), or more general ternary (three phase) solution models (which can be applied to understanding more complex multicomponent systems) that are probably the norm for most biological membraneless organelles (Lee et al., 2013). These mathematical models are well reviewed (Brangwynne et al., 2015, Shin and Brangwynne, 2017).

In these more physiological complex coacervation systems, liquid: liquid phase separation is probably driven both by homotypic interactions between a single type of polymer, and by heterotypic interactions (e.g. protein #1: protein #2 or protein: RNA interactions). The existence of such complex systems, allows for: i) the cooperative interaction of the different polymers in forming the phase separation at lower polymer concentrations; and ii) the existence of membraneless organelles with internal structures comprised of shells, cores, or multiple sub-droplets.

As discussed below, a simple and intuitive example of cooperative interaction is where the introduction of small quantities of RNA can bind multiple FUS proteins through their RNA binding motifs, thereby bringing the protein molecules together at much higher effective/local concentrations than would occur in the absence of the “nucleating” RNA (Berry et al., 2015, Li et al., 2012, Lin et al., 2015, Saha et al., 2016, Zhang et al., 2015).

The molecular diversity of membraneless organelles also allows for the co-existence of multiple phase states in the same condensate. The content of each sub-phase (i.e. shell, core, sub-droplet) is then driven by: i) the relative differences in the component polymers to partition into the different sub-phases based on their relative propensities to form homotypic or heterotypic interactions with each other; and ii) the resulting differences in surface tensions of the component droplets.

Clearly these purely biophysical considerations underpin the structure and function of a diverse group of highly dynamic intracellular organelles. However, as described below using FUS as an example, the precarious balancing act between i) mixed/dispersed; ii) reversible liquid droplet; and iii) stable fibrillar states that may be irreversible in biological systems, and thus places them at risk of causing disease states.

## 6 Potential cellular consequences of pathological phase separation and gelation

Most RNP granules are heterogeneous complexes composed of multiple RNA and protein components that serve as scaffolds or as clients/cargo. As a result, defective function of their component proteins would be anticipated to cause: i) failure of formation of the granule; ii) abnormal partitioning and binding of clients into the granule; iii) abnormal transport of condensed granules; and/or iv) dysregulated release of clients. No examples have *yet* been found of mutations in clinically relevant phase separating proteins that cause defective formation or defective cargo selection. However, it is now clear that disease-causing mutations and disease associated post-translational modifications of several known RNA binding scaffold proteins (e.g. FUS, TDP-43, and hnRNP A1 and A2/B1, and TIA1) can accelerate conversion of these proteins into stable, β-sheet rich, intermolecular hydrogen bonded assemblies (Conicella et al., 2016, Han et al., 2012, Kato et al., 2012, Mackenzie et al., 2017, Molliex et al., 2015, Murakami et al., 2015, Patel et al., 2015, Schmidt and Rohatgi, 2016). The rest of this review therefore focusses on: i) how disease-associated mutations and disease associated post-translational modifications of FUS alter its biophysical properties; and ii) how these changes might affect the function of FUS in neurons.

### 6.1 FUS

FUS is a 526 amino acid heterologous nuclear ribonucleoprotein (hnRNP), and a member of the **F**US, **E**wing sarcoma Breakpoint region 1 (EWS/EWSR1) and **T**ATA box binding protein 15 (TAF15) (**FET**) family of RNA binding proteins (Ratti and Buratti, 2016, Schwartz et al., 2015) (Fig. 2A). It is composed of an N-terminal intrinsically disordered region (residues 1–214), which has reduced amino acid content diversity (i.e. is a low complexity (LC) domain), and which contains multiple glutamine, glycine, serine and tyrosine (QGSY) repeats. In its middle and C-terminal domains, FUS has a well-conserved RNA recognition motif (RRM), a zinc finger domain, and two domains that are enriched in arginine, glycine, glycine (RGG) motifs (Ratti and Buratti, 2016, Schwartz et al., 2015). Finally, FUS contains an atypical proline tyrosine nuclear localisation sequence (PY-NLS) (Dormann et al., 2010, Dormann et al., 2012, Suarez-Calvet et al., 2016).

**Fig. 4:**
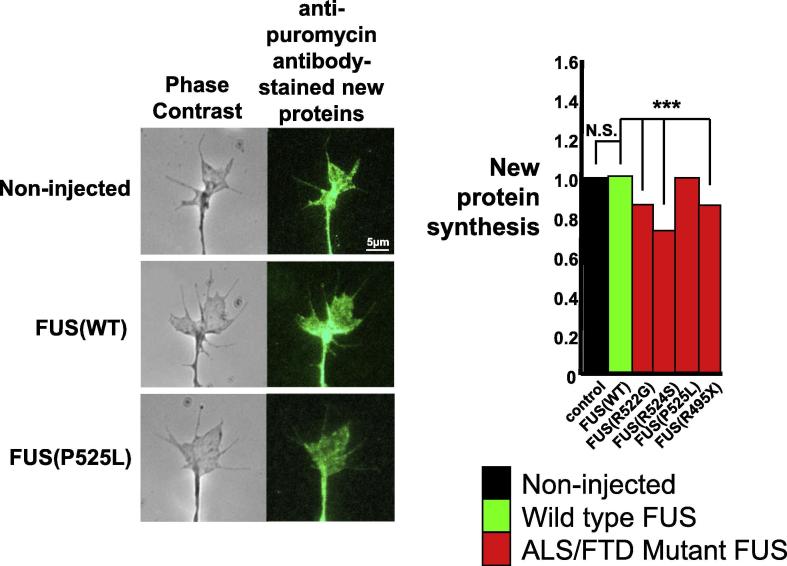
Pathological fALS-FUS mutant FUS condensates have impaired function. By sequestering the machinery of RNA metabolism and translation, the mutant fibrillar condensates cause significant reductions FUS RNP granule function, as measured by reductions in new protein synthesis in axon terminals. New proteins are identified by an assay in which puromycin incorporated into new proteins can be detected using an anti-puromycin antibody. Adapted from Murakami et al., neuron 88: 678, 2015.

**Fig. 5:**
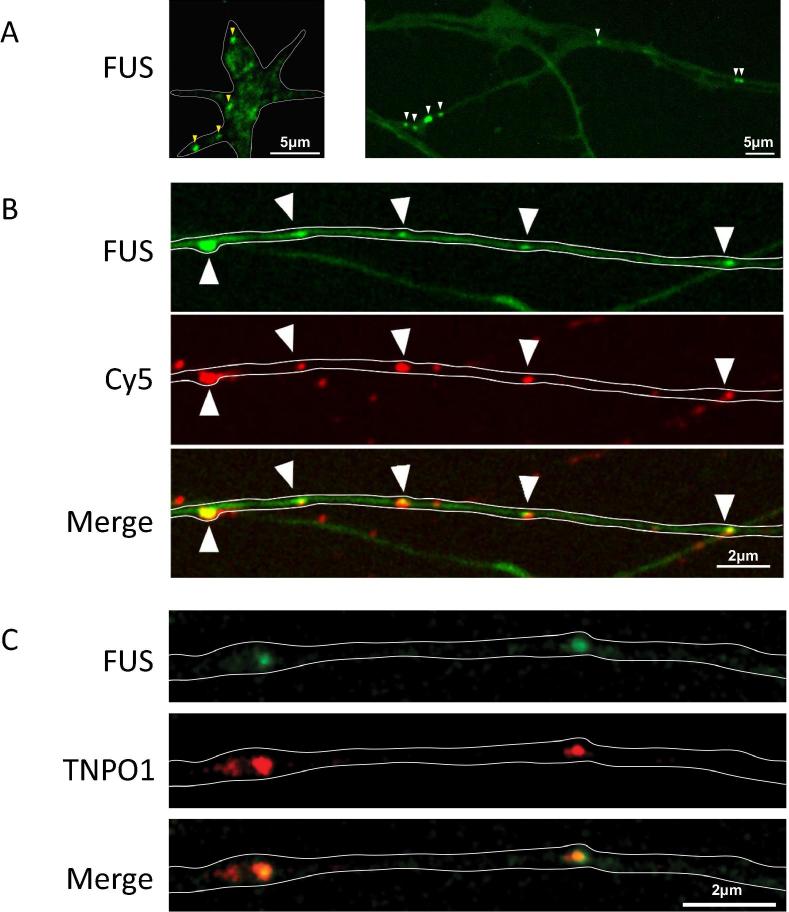
A. FUS RNP granules are detectable in axon terminals and axons using GFP labelled anti-FUS antibody is (arrowheads). B. FUS RNP granules in axon terminals colocalise with markers of RNA (Cy5). C. FUS and TNPO1 colocalise in axons and axon terminals expressing endogenous FUS and Cherry tagged TNPO1. Adapted from Murakami et al., neuron 88: 678, 2015 and Qamar et al. Cell, in press, 2018.

FUS is predominantly located in the nucleus, where it plays a crucial role in both DNA and RNA biology, being involved in both DNA repair as well as RNA transcription and processing (Ratti and Buratti, 2016, Schwartz et al., 2015). FUS is also present at much lower abundance in RNP granules in the cytoplasm in axons and dendrites, where it supports regulated local new protein synthesis (Fig. 4, Fig. 5). Nevertheless, even here it plays a crucial role in mRNA and micro-RNA transport and processing. In neurons, many of these cytoplasmic FUS-related RNAs are involved in synaptic biology and neuronal plasticity (Sephton and Yu, 2015). These aspects of FUS biology have been extensively discussed in the literature, and readers are referred to the many recent and excellent reviews on these topics for further details (Dormann and Haass, 2013, Ratti and Buratti, 2016, Sephton and Yu, 2015). FUS is normally post-translationally modified both by asymmetric dimethylation of arginine residues by protein arginine methyl transferases (Rappsilber et al., 2003), and by serine phosphorylation by DNA protein kinase (Deng et al., 2014).

### 6.2 FUS in disease

FUS has an important role in the formation of myxoid/round cell liposarcoma cancers. In these cancers, a chromosomal translocation fuses the 5′ part of FUS to the **C**/EBP **HO**mologous **P**rotein Gene (CHOP). The resulting FUS/CHOP fusion protein likely phase separates in the nucleus, and then recruits chromatin remodelling or transcription factors in the same way that the EWS-FLI1 fusion protein does in Ewing sarcoma (Boulay et al., 2017).

Full-length FUS itself also has important links to neurodegenerative diseases: namely amyotrophic lateral sclerosis (ALS-FUS); frontotemporal dementia (FTLD-FUS) and essential tremor type 4 (Merner et al., 2012). The latter illness appears to be due to frameshift mutations causing nonsense-mediated decay and a loss-of-function effect, which differs considerably from the effects of missense mutations and altered post-translational modification of FUS that lead to ALS and FTLD, which are the focus of the present review.

A small proportion (approximately 1–4%) of familial ALS (fALS) cases arise from missense or frameshift mutations in FUS (>50 mutations have been described to date). Most of these missense mutations and virtually all of the frameshift mutations cluster in the C-terminal region of FUS between residues 495 and 526, near the atypical nuclear localisation sequence (NLS) domain. A much smaller proportion of ALS associated mutations have been identified in the central and N-terminal region, with two mutations (Ser96Del and Gly156Asp) occurring within the LC domain itself (Kapeli et al., 2017, Rainero et al., 2017).

The clinicopathological characteristics of these fALS-FUS cases have been summarised in many recent reviews and will therefore not be discussed further here beyond noting that the pathognomonic neuropathological features of fALS-FUS are the presence of: profound cytoplasmic FUS aggregates; significant depletion of FUS from the nucleus; and occasional intranuclear FUS aggregates. Crucially, these pathological FUS assemblies typically contain normally methylated (i.e. arginine residues are normally asymmetrically dimethylated) FUS, and the aggregates do not contain other FET proteins or transportin (TNPO1) (Hortobagyi and Cairns, 2017, Mackenzie and Neumann, 2016, Neumann et al., 2012, Troakes et al., 2013).

Extensive cytoplasmic and some nuclear deposits of FUS are also present in neurons and glia in a significant proportion (approximately 10%) of apparently sporadic FTLD cases (Hortobagyi and Cairns, 2017, Mackenzie and Neumann, 2016). In these FTLD-FUS cases, there is usually no family history, and sequencing of the FUS gene has usually failed to detect missense or truncation mutations, although 2 FUS mutations (P106L and M254V) have been described in individual patients with FTLD-FUS. However, these mutations have not been shown to co-segregate with the disease, and their association with FTLD-FUS is therefore not fully proven. Two distinct neuropathological FTLD-FUS sub-phenotypes have been described, namely: neuronal intermediate filament inclusion body disease (NIFID) and basophilic inclusion body disease (BIBD). Despite the distinctive neuropathology, these two subtypes of FTLD-FUS do not have clinical phenotypes that are distinguishable. However, the biochemical signature of FUS inclusions in both subtypes of FTLD-FUS is strikingly different from those of FUS inclusions in fALS-FUS. As mentioned above, in fALS-FUS, FUS inclusions do not contain other FET RNA binding proteins, or transportin, and FUS is typically physiologically asymmetrically di-methylated (Neumann et al., 2012, Troakes et al., 2013). In contrast, in FTLD-FUS, the pathological FUS deposits contain EWS, TAF15 and transportin. Furthermore, arginine residues in FTLD-FUS are significantly demethylated, being predominantly comprised of unmethylated FUS or monomethylated argininines (Dormann et al., 2010, Suarez-Calvet et al., 2016). Finally, FUS deposits in FTLD-FUS are occasionally ubiquitinated, whereas in fALS-FUS, FUS is not ubiquitinated.

The mechanism by which the FUS aggregates induce either fALS-FUS or FTLD-FUS has been extensively discussed in recent reviews, with three non-mutually exclusive ideas currently being considered, namely: 1) impairment of the nuclear transcriptional and splicing roles of FUS due to either its presence in the nucleus as pathological aggregates or due to its depletion from the nucleus; 2) loss of the normal cytoplasmic functions of FUS due to its sequestration in pathological assemblies; and 3) toxic gain of function effects arising from the pathological effects of the FUS assemblies such as retention of RNA templates inside pathological aggregates. Recent work using a variety of approaches has begun to allow these possibilities to be examined in finer detail. Thus, recent studies comparing the phenotype of conditional FUS knockout mice with that of mice expressing fALS-FUS mutant FUS suggest that a gain of toxic function is more probable (Sharma et al., 2016). Similarly, recent studies examining the effect of forced ectopic expression of aggregating proteins have shown that toxicity can be mitigated by using strong nuclear localization signals to force expression out of the cytoplasm and into the nucleus, clearly suggest that defects in nucleo-cytoplasmic translocation play a key role (Kim and Taylor, 2017, Matsukawa et al., 2016, Woerner et al., 2016). Tentative support for the possibility that the toxic effect is primarily a gain of toxic function effect in the cytoplasmic compartment, rather than the nuclear compartment, comes from studies of the earliest pathological changes in mice expressing mutant human FUS. In these mice, the earliest changes, seen by electron microscopy, occur in the structure of presynaptic terminals (So et al., 2018). However, this review will focus on the recently emerging insights into the biophysics of FUS and its propensity to undergo phase separation, and the impact of FUS mutations and post-translational modification of FUS on this process. As described below, these emerging experimental results provide compelling molecular insights into how FUS aggregates occur and how they might injure neurons.

## 7 Physiological FUS phase separation

At physiological concentration range of FUS and salt, and at physiological temperature, FUS undergoes phase separation and gelation in a FUS protein concentration-dependent and salt concentration-dependent manner (Han et al., 2012, Kato et al., 2012, Mann and Snowden, 2017, Murakami et al., 2015, Murray et al., 2017, Patel et al., 2015, Qamar et al., 2018). These condensation processes require the presence of the LC domain, and the LC domain can itself phase separate. However, recent work suggests that for FUS, and likely for many other phase separating proteins, non-LC domains also play a critical role. In FUS, binding of RNA to the RRM and zinc finger domains in the C-terminus of FUS dramatically facilitates phase separation, such that FUS phase separation occurs at lower FUS concentrations than in the absence of RNA (Schwartz et al., 2013). More recently, the C-terminus of FUS itself has also been found to cooperate with the LC domain to induce phase separation at lower FUS concentrations (Qamar et al., 2018). This cooperativity arises because the positively charged guanidino side chains on arginine residues form cation-π interactions with free electrons in the aromatic ring of tyrosine residues that are predominantly located at the N-terminus of FUS (including the LC domain) (Figs. . 2A and 6). Similar cation-π interactions are known to occur with Tudor domain containing proteins like SMN (Tripsianes et al., 2011, Zhang et al., 2017), and with other phase separating proteins like DDX4 (Nott et al., 2015) (Fig. 7A). These cation-π interaction-forming proteins contain lysine or arginine as the cation donor, and tyrosine, phenylalanine or tryptophan as the electron donor. There are four biologically important aspects of this cation-π interaction that are worth further inspection.

**Fig. 6:**
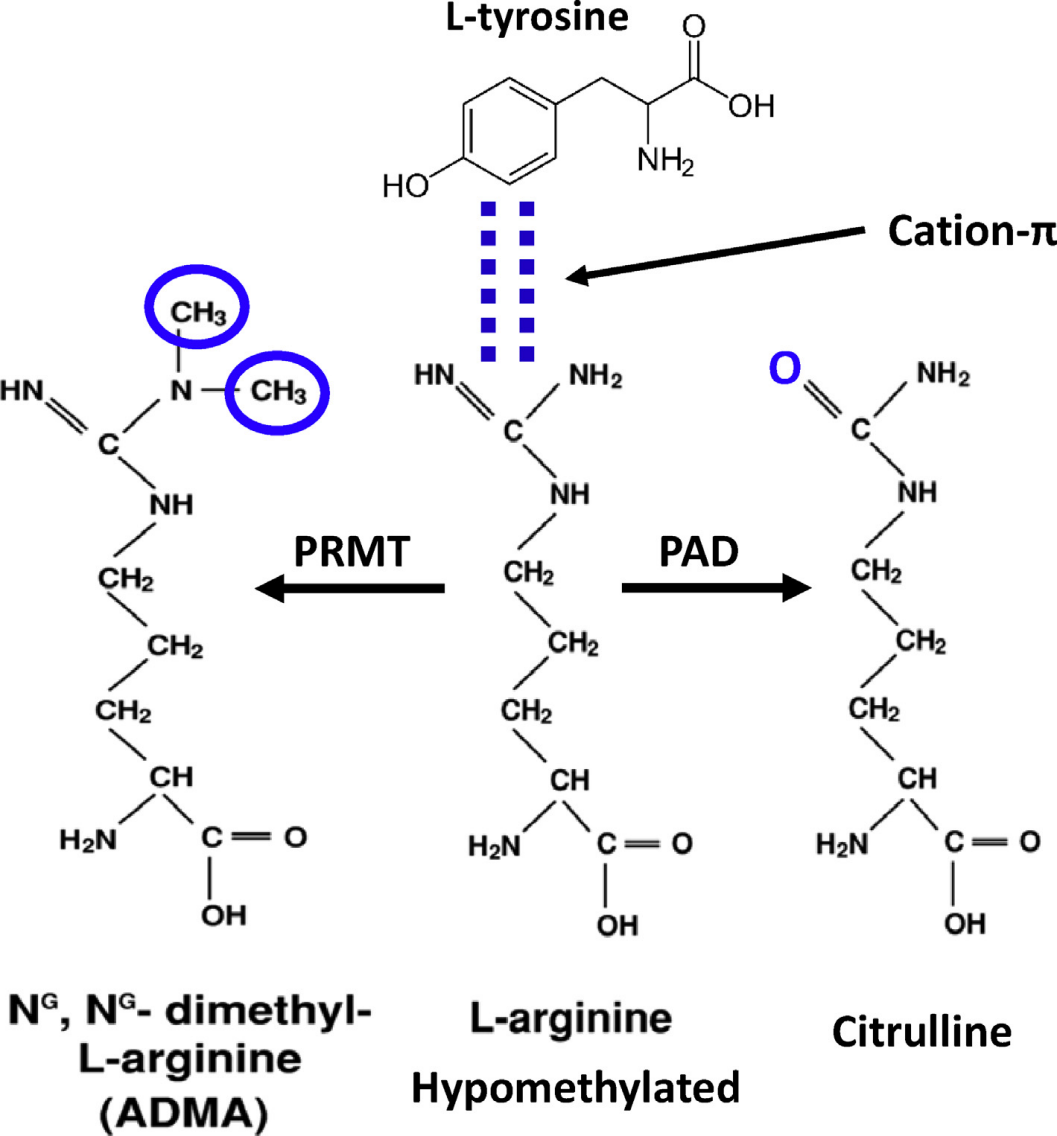
Cartoon depictions of arginine: tyrosine cation-π interactions between the guanidino group in the side-chain of arginine residues, and the aromatic ring of tyrosines. These cation-π interactions can be modulated by arginine methylation by protein arginine methyl transferase enzymes (PRMT), or by conversion to citrulline by protein arginine deiminase (PAD).

**Fig. 7:**
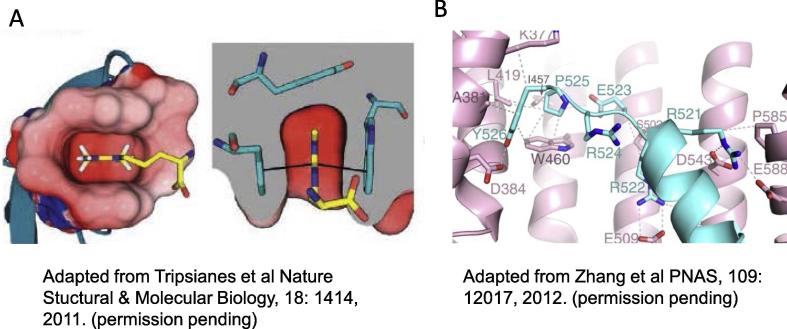
A. Cartoon of an aromatic cage. In some proteins using cation-π interactions, the arginine side-chain fits inside an aromatic cage composed of multiple tyrosine residues in the receptor protein. These aromatic cages are often tuned to preferentially interact with asymmetrically di-methylated arginine (ADMA) residues. Adapted from Tripsianes, Nature Structural and Molecular Biology 18:1414, 2011. B. The terminal helix of FUS interacts closely with the side chains of residues in TNPO1. Many of the C-terminal fALS-FUS mutants (e.g. P525L, and R521G) make key interactions with TNPO1. Adapted from Zhang et al., PNAS, 109:1217, 2012.

The first biologically important feature is the presence of multiple positively charged residues (37 arginines, 14 lysines) and multiple aromatic residues (36 tyrosines, 13 phenylalanines, and 3 tryptophans) that could participate in cation-π interactions. This feature creates the basis for scalable, multivalent, location-independent interactions that drive phase separation, as discussed earlier. The current model for the role of these electrostatic cation-π interactions is that they initiate the phase separation process by promoting FUS: FUS interactions that recruit multiple FUS molecules into restricted volumes wherein there is a much higher local FUS concentrations than in the overall solute (Qamar et al., 2018). This process permits the subsequent formation of stabilising short-range interactions as discussed below. The cooperative effect of RNA on FUS phase separation likely has a similar basis (Schwartz et al., 2013), namely recruitment of multiple FUS proteins into a small volume.

The second biologically important feature is that both arginine and lysine residues are post-translationally modifiable by methylation. Arginine can also be enzymatically deiminated to form citrulline (Fig. 6). These post-translational modifications affect the interaction strength, and thus allow the cation-π interaction to be “tuned”. Citrulllination ablates the cation-π interaction by changing the ketimine moiety into a ketone (Qamar et al., 2018). Methylation weakens the cation-π interactions (Qamar et al., 2018), and in other proteins such as Tudor domain proteins, arginine dimethylation may make the arginine residues more selective only for specific tyrosine residues that exist in three-dimensional pockets, termed “aromatic cages”, comprised by the clustering of aromatic side chains of two or more tyrosine, tryptophan and/or phenylalanine residues (Tripsianes et al., 2011, Zhang et al., 2017) (Fig. 7A). It is unlikely that well-formed aromatic cages are created by tyrosine residues in the intrinsically disordered N-terminus of FUS. However additional studies will be required to determine whether a similar, but transient three-dimensional clustering of specific tyrosines occur in FUS during liquid: liquid phase separation. Post-translational modification of other residues, for instance, phosphorylation of serine by DNA protein kinase (Deng et al., 2014), and theoretically also phosphorylation of threonines and tyrosines by other enzymes, can strongly inhibit phase separation, presumably by disrupting the packing of the LC domains, thereby preventing formation of hydrogen bonded β-sheets (Monahan et al., 2017, Murray et al., 2017).

The third notable feature is that the initial phase separation of FUS that is induced by cation-π interactions is unstable (Qamar et al., 2018). These unstable interactions are likely to be subsequently stabilised by the formation of β-sheet conformations in the LC domain, and by subsequent inter-molecular hydrogen bonding (Qamar et al., 2018). These short-range forces are also scalable. Thus, in liquid droplets, they are present, but not predominant. However, β-sheet derived hydrogen bonding forces are likely to become increasingly significant upon further condensation into denser, hydrogel networks, and especially upon condensation into β-sheet rich, fibrillar assemblies.

**Fig. 8:**
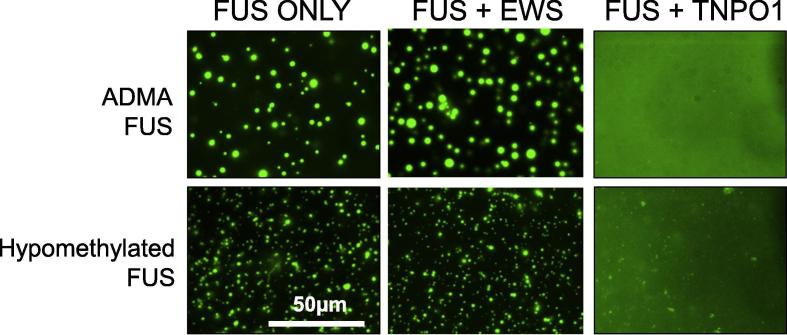
TNPO1 acts as a molecular chaperone for FUS. TNPO1 interacts with FUS to prevent phase separation, and to reverse pre-existing phase separation of both normally methylated ADMA FUS and also pathological hypomethylated FUS. In contrast, the Ewing sarcoma protein (EWS) has no significant impact on FUS phase separation. Adapted from Qamar et al., Cell, in press, 2018.

The fourth notable feature of FUS phase separation is that the arginine rich C-terminus (especially residues 495–526) bind molecular chaperones. One such chaperone is transportin 1 (TNPO1; karyopherin beta-2 (KapBeta2), which binds to the atypical proline tyrosine nuclear localisation sequence (PY-NLS) (Fig. 7B) (Dormann et al., 2010, Dormann et al., 2012, Monahan et al., 2017, Suarez-Calvet et al., 2016). Binding of FUS to TNPO1 supports the shuttling of FUS from the cytoplasm into the nucleus. However, recent work has uncovered a role for TNPO1 in maintaining FUS in a dispersed state and melting pre-existing FUS droplets and gels. TNPO1 can both block FUS phase separation and melt preassembled FUS condensates (Monahan et al., 2017, Qamar et al., 2018). Crucially, TNPO1 is not only expressed at the nuclear pore, but is also expressed in axons and dendrites (Chang and Tarn, 2009, Twyffels et al., 2014), where it is able to modulate the assembly and relaxation of FUS condensates (Qamar et al., 2018) (Fig. 8). By altering the biophysical state of FUS in RNP granules, and thus sequestration/release of FUS-bound mRNAs in the granule, TNPO1 can modulate local RNA translation in axon terminals (Qamar et al., 2018).

### 7.1 Pathological FUS phase separation

As noted earlier, pathological deposits of FUS are prominent neuropathological features of both familial ALS (fALS FUS) and of sporadic FTLD (FTLD-FUS).

### 7.2 fALS-FUS

The above considerations provide a partial insight into how missense and C-terminal truncating frameshift mutations in FUS might promote assembly of FUS into stable fibrillar assemblies that impede FUS function in the ways described earlier (i.e. loss of nuclear functions regulating transcription and splicing, and/or loss of cytoplasmic functions in RNA transport, translation and metabolism).

Only a very small proportion of mutations associated with fALS-FUS occur within the FUS LC domain (FUS Ser96Del and Gly156Asp). While the precise biophysics has not yet been fully worked out, these mutations directly increase the propensity of the LC domain to phase separate, and then form hydrogels (Murakami et al., 2015, Patel et al., 2015). Thus, wild-type FUS LC domain can be cycled between dispersed and phase separated states multiple times (>5) before the process fatigues and the assemblies no longer relax back to the dispersed state after gelation. In contrast, the mutant LC domains have been experimentally shown to fatigue and fail to reverse back to dispersed states after only 1–2 cycles (Murakami et al., 2015).

Most fALS-FUS mutations, however, occur outside the LC domain (Dormann et al., 2012, Murakami et al., 2015, Suarez-Calvet et al., 2016). Mutations affecting residues 495–526 in the C-terminus of FUS likely have two effects. First, they directly increase the propensity of the mutant protein to phase separate and then gelate (Murakami et al., 2015). Second, the fALS-FUS mutations in this region affect residues within the 2.5 turn α-helix of FUS that makes direct contact with TNPO1 (Fig. 7B). Disruption of these contacts reduces the normal binding affinity (Kd = 9.5 nM) by up to 9-fold (Niu et al., 2012, Zhang and Chook, 2012). This double effect likely accounts for why fALS-FUS mutations in the C-terminus are associated with considerably more aggressive ALS phenotypes than mutations in the central domains and LC domains.

The mutations outside the LC domain, but not near the PY-NLS sequence have not been studied in detail. However, they probably also affect FUS phase behaviour either by changing the intrinsic phase separation propensity of FUS and/or by altering the interaction of FUS with nucleating ligands such as RNA and other ribonucleoproteins that bind to the central zinc finger and RRM domains.

### 7.3 FTLD-FUS

Glial and neuronal cytoplasmic and nuclear FUS aggregates are also a pathological feature of some cases of FTLD. Intriguingly, most cases, perhaps even all cases of FTLD-FUS are associated with wild-type FUS gene sequences. Moreover, in contrast to FUS inclusions in fALS-FUS, the FUS inclusions in FTLD-FUS coexist with EWS, TAF15 and TNPO1 (and possibly several other proteins). Importantly, while arginine residues in fALS-FUS-associated mutant FUS appears to be normally asymmetrically dimethylated (ADMA FUS), the arginine residues in FUS within the FTLD-FUS inclusions are significantly demethylated (HYPO FUS) (Dormann et al., 2012, Hortobagyi and Cairns, 2017, Mackenzie and Neumann, 2016, Suarez-Calvet et al., 2016).

The underlying mechanisms leading to demethylation of FUS are not presently clear. However, the hypomethylation of arginines on FUS has two direct effects.

First, hypomethylated or unmethylated FUS has dramatically increased binding affinity for the chaperone TNPO1 (Dormann et al., 2012, Monahan et al., 2017, Suarez-Calvet et al., 2016), which might result in failure of TNPO1 to release FUS into the nucleus, and thus causes FUS accumulation in the cytoplasm.

Second, FUS with unmethylated or hypomethylated arginines has an increased intrinsic propensity to both phase separate and gelate (Qamar et al., 2018). This propensity is dependent upon the number of non-dimethylated arginine residues. The underlying biophysical mechanism is presently unknown. One possibility is that the unmethylated guanidino side chains of un-methylated arginine residues may simply make higher affinity interactions with the aromatic rings of the N-terminal tyrosine residues. Another possibility is that these unmethylated arginine side chains may make promiscuous interactions with the aromatic rings of additional N-terminal tyrosine residues that do not normally participate in cation-π interactions during physiological condensation of FUS (e.g. because they are not within some putative “aromatic cage-like” structure) (Qamar et al., 2018). Dissection of these possibilities, which has direct relevance for the development of therapies to mitigate these un-methylated arginine interactions, will require additional work, including perhaps solid-state NMR studies of FUS in different methylation states.

## 8 Discussion

The exciting work reviewed here describes a rapidly emerging area of cell biology related to the function of membraneless organelles such as nucleoli, P bodies, stress granules, neuronal transport granules and other RNP granules. The basic biophysics of how these phase separation and gelation events occur and the effect of disease-causing mutations/post-translational modifications on these events is now becoming clearer.

The next phase of work will need to focus on understanding how normal assembly and relaxation/disassembly of condensates is physiologically regulated in response to cellular metabolic state.

This future work may also provide some tractable molecular targets for novel treatments for diseases associated with abnormal phase separation of intrinsically disordered proteins, such as FUS, TDP-43 and other RNA binding proteins.
